# Zika virus shedding in the stool and infection through the anorectal mucosa in mice

**DOI:** 10.1038/s41426-018-0170-6

**Published:** 2018-10-17

**Authors:** Chunfeng Li, Yong-Qiang Deng, Shulong Zu, Natalie Quanquin, Jingzhe Shang, Min Tian, Xue Ji, Na-Na Zhang, Hao-Long Dong, Yan-Peng Xu, Ling-Zhai Zhao, Fu-Chun Zhang, Xiao-Feng Li, Aiping Wu, Genhong Cheng, Cheng-Feng Qin

**Affiliations:** 1 Center for Systems Medicine, Institute of Basic Medical Sciences, Chinese Academy of Medical Sciences & Peking Union Medical College, Beijing, 100005, China; 2 Department of Virology, State Key Laboratory of Pathogen and Biosecurity, Beijing Institute of Microbiology and Epidemiology, Beijing, 100071 China; 3 Suzhou Institute of Systems Medicine, Suzhou, Jiangsu 215123 China; 4 Guangzhou Eighth People’s Hospital, Guangzhou Medical University, Guangzhou, 510060 China; 5 CAS Key Laboratory of Infection and Immunity, Institute of Biophysics, Chinese Academy of Sciences, Beijing, 100101 China; 6 University of Chinese Academy of Sciences, Beijing, 100049 China; 7 Department of Microbiology, Immunology and Molecular Genetics, University of California, Los Angeles, CA 90095 USA

## Abstract

Zika virus (ZIKV) has elicited global concern due to its unique biological features, unusual transmission routes, and unexpected clinical outcomes. Although ZIKV transmission through anal intercourse has been reported in humans, it remains unclear if ZIKV is detectable in the stool, if it can infect the host through the anal canal mucosa, and what the pathogenesis of such a route of infection might be in the mouse model. Herein, we demonstrate that ZIKV RNA can be recovered from stools in multiple mouse models, as well as from the stool of a ZIKV patient. Remarkably, intra-anal (i.a.) inoculation with ZIKV leads to efficient infection in both *Ifnar1*^*−/−*^ and immunocompetent mice, characterized by extensive viral replication in the blood and multiple organs, including the brain, small intestine, testes, and rectum, as well as robust humoral and innate immune responses. Moreover, i.a. inoculation of ZIKV in pregnant mice resulted in transplacental infection and delayed fetal development. Overall, our results identify the anorectal mucosa as a potential site of ZIKV infection in mice, reveal the associated pathogenesis of i.a. infection, and highlight the complexity of ZIKV transmission through anal intercourse.

## Introduction

Zika virus (ZIKV), a flavivirus that resurged in recent outbreaks in the Americas, was first isolated from a monkey in the Zika forest of Uganda in 1947^1^. After the first confirmed human infection in 1964^2^, ZIKV spread silently through Africa and Asia over the next 40 years. The first reported outbreak of ZIKV was on Yap Island in 2007, followed by a severe epidemic in 2013 in French Polynesia, where there was an increased incidence of Guillain-Barré syndrome (GBS) noted in ZIKV-positive cases^3^. The ZIKV outbreak that began in Brazil in 2015 was associated with severe birth defects such as microcephaly, which was later confirmed in both animal models and clinical investigations^4–7^.

Similar to other related flaviviruses, such as dengue virus (DENV) and yellow fever virus (YFV), ZIKV is primarily transmitted through the bite of infected *Aedes* mosquitoes^8^. Interestingly, ZIKV has also been detected in semen^9^ and the vaginal swabs of pregnant women^10^. This would explain the reported cases of sexual transmission in humans^11^. Among these cases, ZIKV was primarily transmitted from infected males to uninfected females^12–14,^ however, there was one case of transmission from an infected man to his uninfected male partner through anal intercourse^15^. Unprotected anal intercourse is the mode of transmission for ~7.4% of all human sexually transmitted infections^14^, which supports the possibility that the anorectal mucosa serves as an entry site for ZIKV infection.

Duggal et al. observed that ZIKV could be transmitted efficiently through male-to-female intercourse in an immunodeficient mouse model, as the virus is shed for prolonged periods in the seminal fluid^16^. Furthermore, Yockey et al. found that ZIKV intravaginal infection of pregnant mice, even in immunocompetent mouse strains, can lead to in utero infection of the offspring^17^. These findings indicate that the vaginal tract mucosa can serve as an entry site for ZIKV infection. However, whether the anorectal mucosa can also support viral replication, and the potential systemic effects from that infection, remain largely unknown.

ZIKV is also unique among flaviviruses in that high concentrations are present in multiple secretions and body fluids of an infected host. In guinea pigs and monkeys, intranasal inoculation with ZIKV leads to systemic infection. Interestingly, even close contact is sufficient for ZIKV to be transmitted between guinea pigs^18^. This can be partially explained by the fact that ZIKV is detectable in the saliva and tears of humans, monkeys, and guinea pigs^18–20^. ZIKV can also be isolated from the urine and breast milk^21,22^. However, whether ZIKV can be recovered from fecal specimens needs to be investigated.

In this study, we show that ZIKV can be detected in the feces of infected mice and a human, suggesting that stool samples could be used for surveillance of ZIKV infection in humans or sentinel animals. Importantly, ZIKV can infect mice through the anorectal mucosa to cause viremia with subsequent testicular damage in ZIKV-infected male *Ifnar1*^*−/−*^ mice, as well as congenital defects in the offspring of wild-type pregnant C57BL/6 (C57) mice treated with anti-Ifnar1 antibody. This work describes a novel mouse model for examining an under-studied route of ZIKV transmission and pathogenesis.

## Results

### ZIKV is shed into the stool of infected mice

**Fig. 1:**
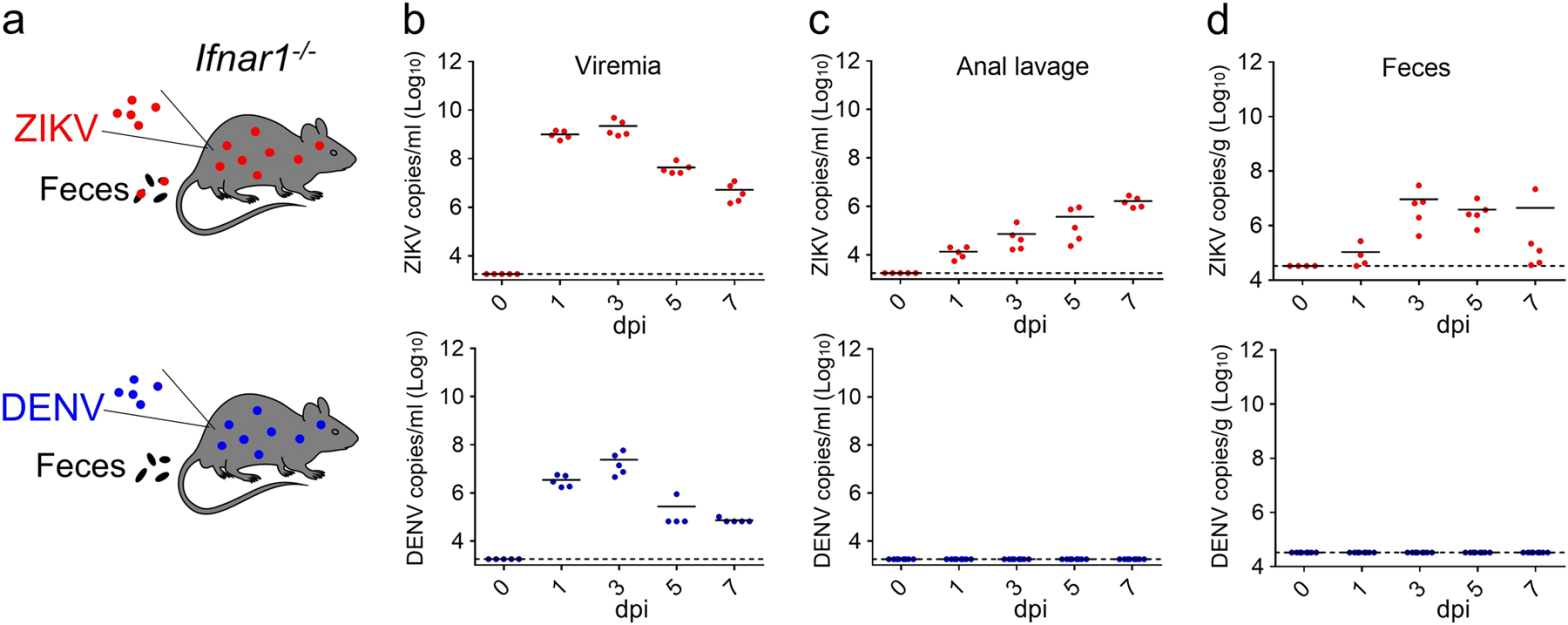
ZIKV is shed into the feces of infected *Ifnar1*^−/−^ mice **a** Experimental outline for ZIKV infection. Mice were infected with 10^5^ PFU of either ZIKV (GZ01 strain) or DENV-2 (43 strain) by the i.p. route. **b**–**d** RNA in the sera (**b**), anal lavage fluid (**c**), and feces (**d**) from ZIKV- or DENV-infected *Ifnar1*^−/−^ mice was analyzed at the indicated time points by qRT-PCR using primers specific for each virus. (*n* ≥ 5 per group)

To assess whether ZIKV is shed into the stool of an infected host, we initially selected *Ifnar1*^−/−^ mice as a well-established model of ZIKV infection^23^. DENV, a closely related flavivirus, was also tested for comparison (Fig. 1a). Upon intraperitoneal (i.p.) infection with a contemporary Asian strain of ZIKV (GZ01, 10^5^ PFU/mouse), all *Ifnar1*^−/−^ mice developed sustained viremia, with peak titers at 3 days post-infection (dpi) (Fig. 1b). The average and peak viremia levels in DENV-infected mice were much lower than what was measured in ZIKV-infected mice (Fig. 1b). Elevated levels of ZIKV RNA were also detected in the anal lavage fluid and fecal samples of ZIKV-infected animals, even at 7 dpi (Fig. 1c, d). Remarkably, DENV RNA was not detected in either anal lavage or fecal specimens (Fig. 1c, d). When we repeated this experiment using another historical ZIKV strain, FSS13025, viral RNA was also detected in the feces and anal lavage fluid (Fig. S1). The viral genome could be amplified from the fecal sample by RT-PCR, and routine sequencing did not identify any meaningful nucleotide variations (data not shown). Furthermore, consistent with previous findings^23^, both ZIKV and DENV could be detected in all tested organs at 5 dpi when infection occurred by the i.p. route (Fig. S2). Interestingly, ZIKV and DENV RNA were abundant in the small intestine and rectum (Fig. S2).

**Fig. 2:**
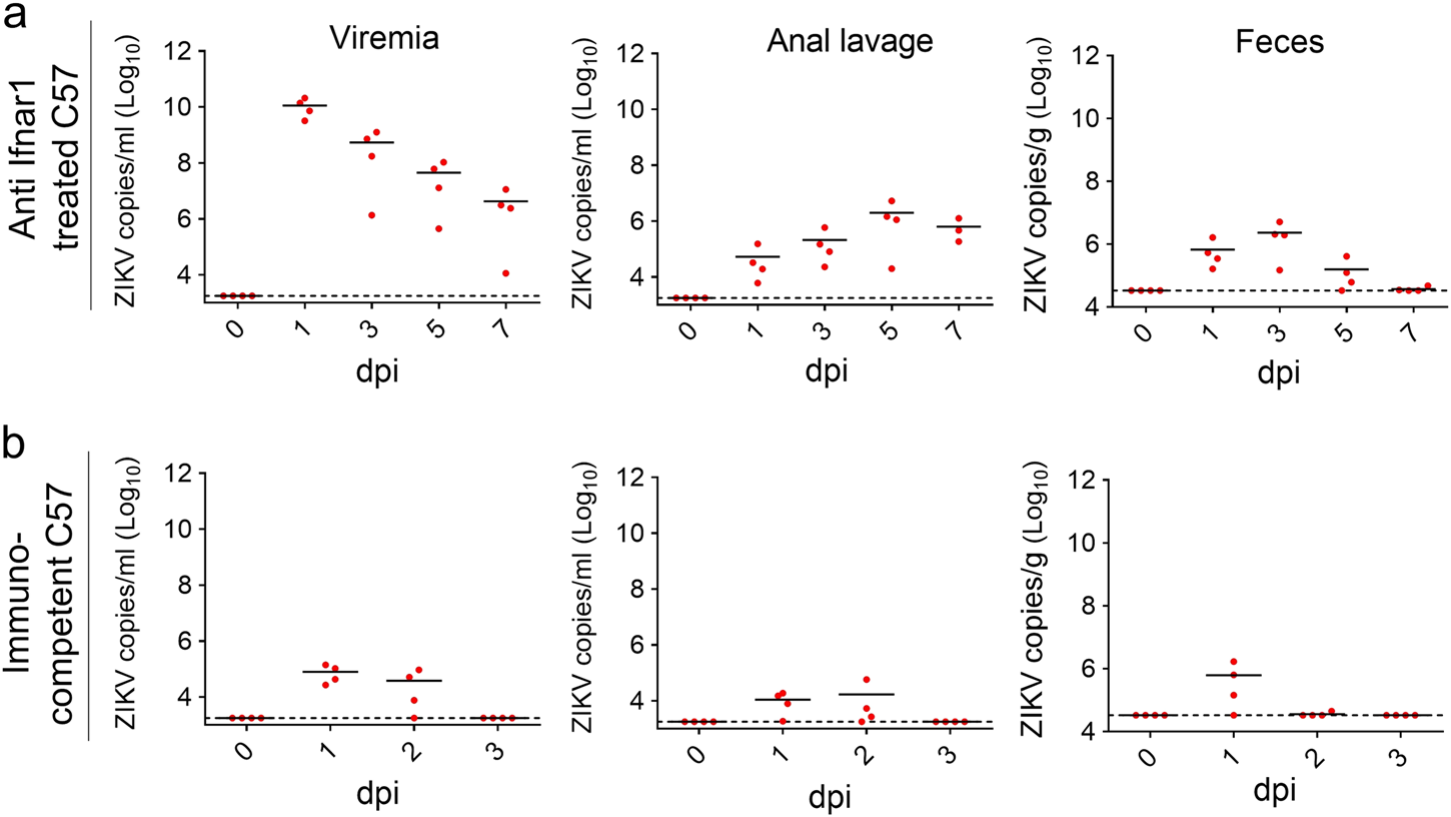
ZIKV is shed into the feces of infected immunocompetent mice **a**–**b** 3–4-week-old C57 mice were pretreated with anti-Ifnar1 antibody (**a**) or mock-treated (**b**) for 12 h and then infected with ZIKV (GZ01 strain, 10^6^ PFU/mouse) by the i.p. route. ZIKV RNA copies in the blood, anal lavage and feces at the indicated times were quantified by qRT-PCR (*n* = 4 per group)

We next tested whether ZIKV could also be recovered from the feces of an infected immunocompetent strain of mice. As previously described^24^, C57 mice pretreated with anti-Ifnar1 antibody for 12 h were subsequently infected with ZIKV strain GZ01 (10^6^ PFU/mouse) by the i.p. route. Sustained viremia was readily detected at 1, 3, 5, and 7 dpi, at which time viral RNA was also detectable in both the anal lavage fluid and feces, albeit at a lower magnitude (Fig. 2a). We then tested whether C57 mice that were not pretreated with anti-Ifnar1 antibody^25^ had similar results. ZIKV RNA was measurable in both the blood and anal lavage fluid at 1 and 2 dpi, while virus was only detected in the feces at 1 dpi (Fig. 2b). To support our findings, we were fortunate to obtain a stool sample from one patient^22^ during the acute period of ZIKV infection. A qRT-PCR assay of the sample showed high levels of ZIKV RNA (~2 × 10^5^ copies/g) 3 days after the onset of fever, when urine samples were also positive for ZIKV RNA^22^. ZIKV infection triggered the upregulation of multiple cytokines in humans during acute phase of infection that we were able to characterize by Luminex assay, including IP-10, IL-5, GM-CSF, TNF-α, IL-15, IL-10, MIP-1α, IFN-γ, IL-6, IL-1RA, IL-2, MIG, IFN-α, IL-2R, and IL-4 (Fig. S3). Altogether, our studies confirm that ZIKV is shed into the feces of both mouse models and humans during active infection.

### ZIKV intra-anal inoculation leads to systemic infection in mice

**Fig. 3:**
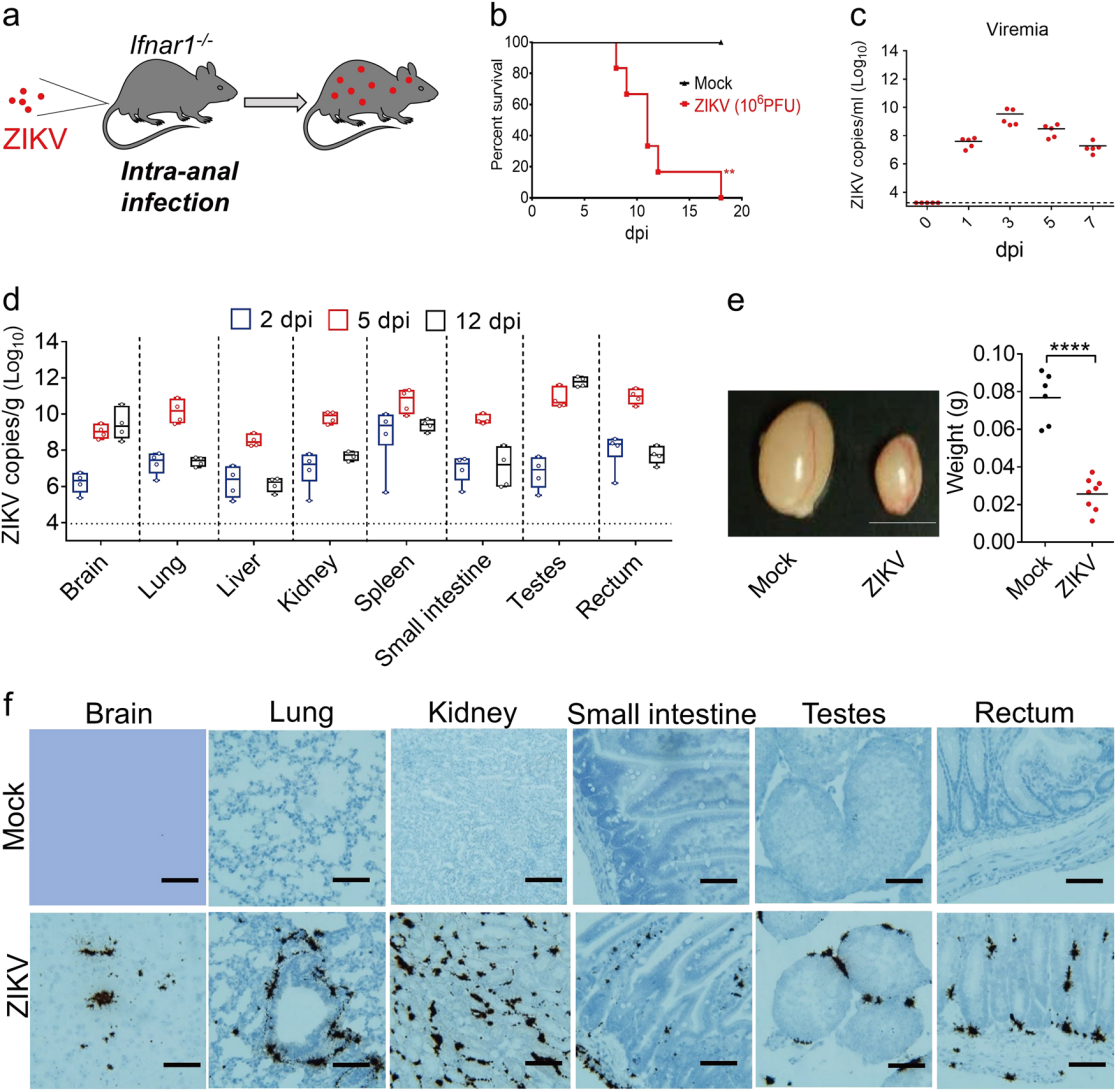
ZIKV intra-anal inoculation leads to systemic infection in mice **a** Illustration of the experiment. 3–4-week-old male *Ifnar1*^−/−^ mice were infected with ZIKV (10^6^ PFU/mouse) by the i.a. route (**a**–**f**). **b** The survival rate of the infected *Ifnar1*^−/−^ mice (*n* = 6 per group). **p < 0.01, log-rank (Mantel–Cox) test. **c** Viral RNA copies in sera were measured at the indicated time points by qRT-PCR assay (*n* = 5 per time point). **d** ZIKV RNA recovered from the brain, lung, liver, kidney, spleen, testes, small intestine, and rectum were quantified by qRT-PCR (*n* = 4 per group). **e** The size and weight of the testes from uninfected or ZIKV anally-infected *Ifnar1*^−/−^ mice were measured at 15 dpi (*n* = 6, mock; *n* = 8, ZIKV). Scale bar = 0.5 cm. ****p < 0.0001, unpaired Student’s *t*-test. **f** RNA in situ hybridization of organ tissues from mock-infected or ZIKV anally-infected *Ifnar1*^−/−^ mice with a ZIKV-specific probe at 5 dpi. Scale bar represents 100 μm. The data in (**f**) are representative of two independent experiments with at least two animals per group

The excretion of ZIKV into the feces also highlights the potential risk of ZIKV transmission through anal intercourse. We sought to clarify whether the anorectal mucosa of mice could serve as an entry site for ZIKV infection. We injected *Ifnar1*^−/−^ mice by the intra-anal (i.a.) route with ZIKV strain GZ01 (10^6^ PFU/mouse)^22^ (Fig. 3a). All infected mice developed the characteristic symptoms of ZIKV systemic infection, including weight loss beginning at 6 dpi (Fig. S4a) followed by inactivity, encephalitis, hindlimb paralysis (Fig. S4b), and death between 8–18 dpi (Fig. 3b). To examine the lethality of ZIKV infection when the virus was administered by the i.a. route, we infected *Ifnar1*^−/−^ mice with 10^4^ or 10^5^ PFU doses. The results indicate mortality rates of 20 and 67% at 10^4^ and 10^5^ PFU, respectively (Fig. S5a). Even at 10^4^ PFU, the virus could be recovered from the serum and all tested organs, suggesting that i.a. infection with ZIKV is highly efficient in *Ifnar1*^−/−^ mice (Fig. S5b, c). ZIKV infection through the i.a. route resulted in detectable viremia at 1 dpi, which peaked at 3 dpi before falling at 5 to 7 dpi (Fig. 3c). To identify the tissue tropisms for ZIKV when transmitted by the i.a. route, multiple organs were collected at 2, 5, and 12 dpi and subjected to qRT-PCR analysis. As shown in Fig. 3d, ZIKV RNA was detected in all tested organs, including the brain, lung, liver, kidney, spleen, small intestine, testes, and rectum. Interestingly, ZIKV RNA levels in the brain and testes continued to increase until 12 dpi, while the levels peaked at 5 dpi in other organs. At early time points (2 dpi), ZIKV was at its highest levels in the spleen and rectum. By 5 dpi, the level in the testes had risen significantly, surpassing the level in the spleen and rectum, where the levels began to drop. Although not reaching the high levels seen in the testes, the ZIKV burden in the brain continued to rise through day 12 (Fig. 3d). Plaque assays with tissue homogenates collected at 5 dpi showed the presence of high concentrations of infectious ZIKV (Fig. S6a). Interestingly, we were also able to establish ZIKV infection by the i.p. route in naive *Ifnar1*^−/−^ mice using the rectal wash fluid from i.a.-infected mice at 5 dpi, confirming the prolonged presence of infectious ZIKV in the anorectal environment. The recipient mice died by 10 dpi, with viremia from 1 to 8 dpi that peaked at 3 dpi (Fig. S6b, c).

ZIKV infection by the i.p. route has been shown to lead to testicular damage and infertility in mouse models^26,27^. Similarly, we noted that the testes of mice infected by the i.a. route with ZIKV were much smaller than those of mock-infected mice at 15 dpi (Fig. 3e). Furthermore, H&E staining revealed severe damage to the structure of the testes, with Leydig cell fibrosis in ZIKV-infected mice (Fig. S7). ZIKV infection was similarly associated with inflammatory cell infiltration and vascular cuffing in the mouse brain and mild inflammation in the rectum (Fig. S7). More importantly, our RNA scope assay showed that ZIKV RNA was present in the testes, brain, rectum, lung, kidney, and small intestine at 5 dpi, but not in mock-infected samples (Fig. 3f). Interestingly, ZIKV-specific RNA was found in the Leydig cells of the testes, which is in agreement with previous findings in guinea pigs and mice^18,26^.

We further validated the above result in immunocompetent C57 mice. Upon i.a. infection, ZIKV RNA could be detected in the blood of mice at 1 and 2 dpi, but not at 3 dpi (Fig. S8a). Furthermore, ZIKV could be found in all inspected organs except for the brain. The highest burden of ZIKV was seen in the spleen, rectum, and anal canal at 1 dpi (Fig. S8b). The above results show that i.a. inoculation of ZIKV in immunocompetent and *Ifnar1*^−/−^ mice leads to systemic infection of almost all organ systems, with resulting testicular damage and inflammation in the brain and rectum in *Ifnar1*^−/−^ mice.

### ZIKV infects and triggers innate immune responses in human colon cells

**Fig. 4:**
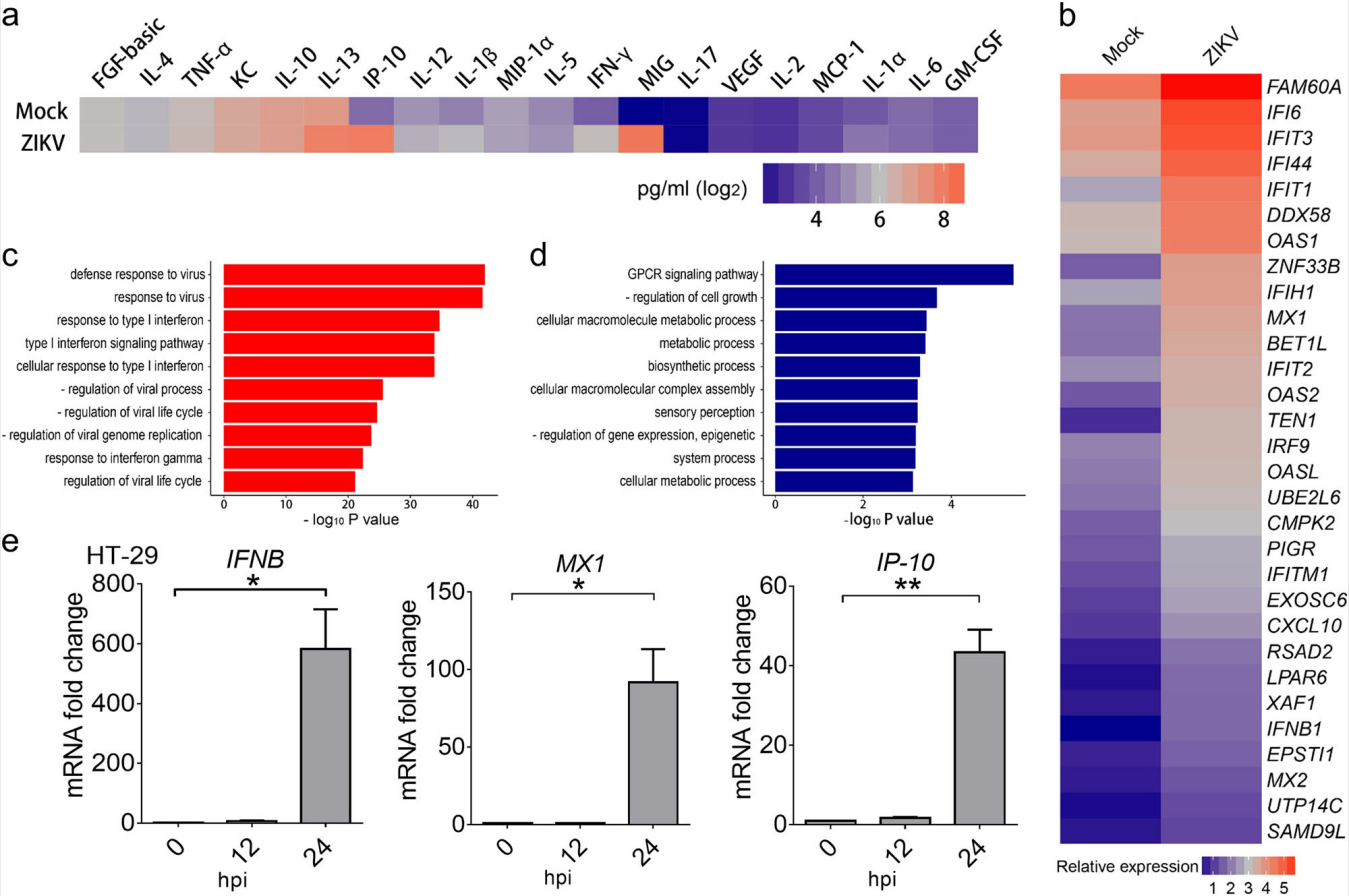
ZIKV infects and triggers innate immune responses in human colon cells **a** Expression levels of cytokines and chemokines in sera from ZIKV anally-infected *Ifnar1*^−/−^ mice (10^6^ PFU/mouse), analyzed by Luminex assay at 7 dpi. The average expression levels of cytokines from 3 mice are shown. **b** Relative expression levels of the 30 innate immune response genes in human colon cells (HT-29) most upregulated by ZIKV infection. The cells were infected with ZIKV (GZ01 strain, MOI 0.5) for 24 h, and total RNA was extracted and analyzed by RNA-seq. **c**, **d** Pathways for the up- (**c**) or down- (**d**) regulated genes enriched by gene ontology pathway analysis. "-" means "negative". **e** The mRNA expression levels of *IFNB, MX1*, and *IP-10* in ZIKV-infected (GZ01 strain, MOI 1) HT-29 cells were measured by qRT-PCR at the indicated time points. All data are shown as the mean ± SEM. *p < 0.05, **p < 0.01, unpaired Student’s *t*-test

To profile the specific immune responses triggered by i.a. infection of mice with ZIKV, we assayed their ZIKV-specific antibody kinetics. We can see that ZIKV-specific IgM and IgG antibodies were detected at 7 dpi in mice infected by the i.a. route (Fig. S9). Furthermore, Luminex assay showed the upregulated expression of multiple cytokines and chemokines, including IFN-γ, IP-10, MIG, IL-13, and IL-12 (Fig. 4a).

To help characterize the innate response to ZIKV in the anorectal mucosa of humans, we performed infections in two human colon epithelial cell lines, HT-29 and SW620. As shown in Fig. S10, ZIKV efficiently infected both groups. RNA-seq analysis of HT-29 cells revealed that ZIKV infection caused the upregulation of *IFNB* and several innate immune response genes, including *FAM60A, IFI6, DDX58, OAS1, MX1*, and *IP-10* (Fig. 4b and Fig. S11a). Gene ontology analysis showed that many genes associated with antiviral activity or the type I interferon signaling pathway were upregulated (Fig. 4c), highlighting the importance of the innate immune response in the clearance of ZIKV. Interestingly, many genes related to cell growth and metabolism were downregulated (Fig. 4d), consistent with the gene expression profiles seen in ZIKV-infected human skin cells and neonatal brains^4,28^. These results were confirmed by qRT-PCR analysis, showing upregulated expression of *IFNB, MX1, IP-10, CH25H, ISG15, OASL, IFITM3, RIG-I*, and *TRIM21* following ZIKV infection at 24 hpi but not 12 hpi (Fig. 4e and Fig. S11b–g), in agreement with previous findings in A549 cells^29^. Interestingly, our analysis also revealed several previously unidentified ZIKV-induced genes (ZIGs) in human colon cells, such as *FAM60A, BET1L*, and *LPAR6*.

### Intra-anal infection of pregnant mice with ZIKV leads to transplacental transmission and impaired fetal development

**Fig. 5:**
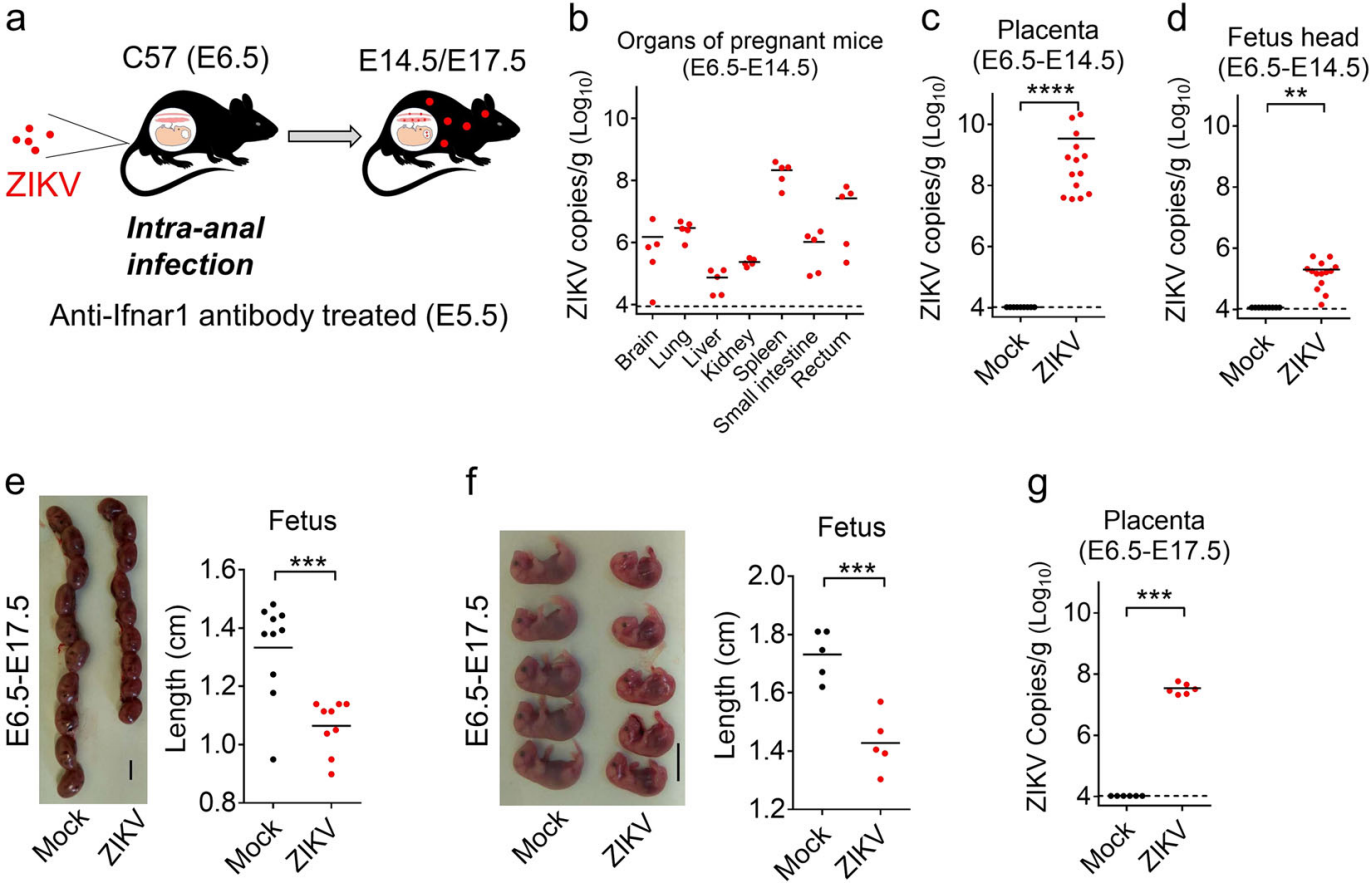
Intra-anal infection of pregnant mice with ZIKV leads to transplacental transmission and impaired fetal development **a** Illustration of the experiment. 7–8-week-old C57 mice were mated, and pregnant mice were infected i.a. by ZIKV (10^6^ PFU/mouse) at E6.5 after treatment with anti-Ifnar1 antibody at E5.5. Mice were euthanized at E14.5 (E6.5-E14.5) or E17.5 (E6.5-E17.5). **b**–**d** ZIKV RNA copies recovered at E14.5 (8 dpi) from tissues of pregnant mice, including the brain, lung, liver, kidney, spleen, small intestine, and rectum (*n* = 5 per group) (**b**), or the offspring of 2 representative pregnant mice including the placenta (**c**) or the fetal head (**d**) were quantified by qRT-PCR (*n* = 9, Mock; *n* = 14). **e**, **f** Condition and length of individual fetuses at E17.5 (**e** *n* = 10 Mock; *n* = 9 ZIKV; **f** *n* = 5 per group). The size of each fetus within the amniotic sac is shown in (**e**), while (**f**) shows the length measured when the placenta and amniotic tissues are removed. Scale bar = 1 cm. **g** Viral load in the placenta at E17.5 (*n* = 6 per group). Offspring in (**e**–**g**) were from one representative pregnant mouse. **p < 0.01, ***p < 0.001, ****p < 0.0001, unpaired Student’s *t*-test

Finally, we examined the possible effects of maternal i.a. ZIKV infection on the fetus. C57 mice were mated and the dams were subsequently administered anti-Ifnar1 antibody at E5.5, followed by *i.a* inoculation with ZIKV at E6.5 (Fig. 5a). A group was euthanized at E14.5 to measure viral loads from the organs of the adult mice, the placenta, or the fetal head. ZIKV RNA was detected in all tested organs of adult mice, with the highest level found in the spleen (Fig. 5b). Viral loads in the placenta and head of the fetus, as measured by qRT-PCR, were particularly elevated (Fig. 5c, d). Meanwhile, the condition and size of each fetus was measured at E17.5, with the fetuses from ZIKV-infected mice noted to be much smaller than those in the mock-infected group (Fig. 5e, f). ZIKV RNA was also recovered from placental tissue at E17.5 (Fig. 5g). These results show that the i.a. infection of pregnant mice with ZIKV leads to transplacental infection and impaired development of the fetus.

## Discussion

In this study, we examined two separate mouse models to characterize the infectivity and resulting pathogenesis of ZIKV infection by the i.a. route, which is the likely mode of transmission following anal intercourse. Our results showed that i.a. inoculation leads to ZIKV viremia and systemic spread in both immunocompetent and *Ifnar1*^−/−^ mouse strains. In *Ifnar1*^−/−^ mice, ZIKV i.a. infection leads to weight loss, organ inflammation, including significant testicular damage, and death. In pregnant C57 mice treated with anti-Ifnar1 antibody, the viremia following i.a. inoculation of ZIKV also crosses the placenta, resulting in suppression of fetal growth and development.

We made the important discovery that ZIKV can be shed into the stool, even in immunocompetent hosts. Other reports have found ZIKV in high concentrations in multiple body fluids and secretions such as saliva, tears, urine, and semen, with the ability to be transmitted by close contact or sexual activity^9,19,22^. While sexual transmission has not been documented for other flavivirus infections, some members of this family have been recovered from fecal samples of avian hosts. The RNA of Tembusu virus was detected in duck feces and cloacal swabs^30,31^. Infectious particles of West Nile virus (WNV) were isolated from oral and cloacal swab samples of dead birds^32^. The possibility of fecal-oral transmission of ZIKV is unknown, however, our detection of ZIKV in human stool and anal lavage samples does suggest a mechanism for sexual transmission through anal intercourse. It also introduces the possibility of using such patient samples as an additional screening tool for ZIKV diagnosis or epidemiological monitoring.

Although there has been only one reported human case of i.a. transmission to date^15^, the actual incidence of ZIKV transmission through anal intercourse could in fact be quite substantial. Reporting of sexually transmitted infections in general is notoriously low due to factors such as shame, fear, cost, and misinformation^33–35^. ZIKV infection itself is often missed in endemic regions because 80% of cases are asymptomatic^36^. This makes it all the more likely that silent transmission between sexual partners could be occurring, creating new reservoirs of the virus even in areas where mosquito-borne transmission is not prevalent. Our findings underscore the importance of using condoms to prevent ZIKV transmission in individuals who engage in anal intercourse, especially since ZIKV has been detected in seminal fluid for up to 6 months^37^.

Recently, Haddow et al. showed that both intrarectal and intravaginal inoculation of monkeys with ZIKV resulted in a high rate of infection^38^, which is in agreement with our results. In vitro experiments from our lab and others demonstrated that colonic cells (Caco-2, HT-29, and SW620) are susceptible to ZIKV infection^39^. This supports our hypothesis that ZIKV infection can be established in the mucosa of the anorectal canal, which explains the probable mechanism of infection for the reported case of male-to-male human ZIKV transmission. From there, it can spread in the bloodstream and trigger ZIKV-specific humoral immune responses and symptomatic infection, as seen in the single reported human case (subjective fever, myalgia, headache, lethargy, malaise, rash, arthritis, and conjunctivitis). In our experiments with immunocompromised mice, the infection resulted in extensive viral replication in the blood and multiple organs, including the brain, small intestine, testes, and rectum. Moreover, i.a. inoculation of ZIKV in pregnant mice rendered more susceptible to infection using the anti-Ifnar1 antibody resulted in transplacental infection and delayed fetal development. It would be interesting to investigate whether other flaviviruses could also infect intestinal cells and be shed in the stool. Future experiments could also more closely examine the transmissibility of ZIKV in human stool samples. Our knowledge of the pathogenesis of i.a. ZIKV infection in humans is currently limited to the single reported case, and we can only extrapolate from animal models what might occur in potentially uncontrolled infection in immunocompromised humans.

In summary, our data characterize the infectivity and pathogenesis caused by ZIKV i.a. infection using both *Ifnar1*^−/−^ and immunocompetent mice. We demonstrated the particularly high susceptibility of the brain, testes, and gastrointestinal tissues of *Ifnar1*^−/−^ mice to ZIKV infection by this route and that the placenta and fetus remain a target of infection even in immunocompetent strains of mice. Interestingly, we showed that ZIKV RNA can be recovered from the stool, which could potentially be used as a future tool to diagnose or monitor for ZIKV infection. This also highlights the importance of men who are suspected to be carrying ZIKV to avoid unprotected anal intercourse. This route of ZIKV infection represents a new field of study for ZIKV-host pathology, which could impact not only transmission, but possibly future surveillance of ZIKV.

## Materials and Methods

### Human serum and stools

Human serum and stools from healthy individuals and ZIKV-infected patients were collected at the Guangzhou Eighth People’s Hospital, Guangzhou Medical University. This study was performed according to the principles of the Declaration of Helsinki and approved by the Guangzhou Eighth People’s Hospital, Guangzhou Medical University, for the protection of human subjects. All the participants provided written informed consent. The study was approved by the ethics committee of the Guangzhou Eighth People’s Hospital (Reference Number 20160264) according to the medical research regulations of China.

### Mice and ethics statement

*Ifnar1*^−/−^ mice (IFN-α/β receptor deficient) were kindly provided by Professor Qi-Bin Leng (Institut Pasteur of Shanghai, Chinese Academy of Sciences, China). C57 mice were purchased from the Vital River Laboratory (Beijing, China) and bred in our core animal facility. All animal experiments were performed according to the standard operating protocol (SOP) issued by the Animal Experiment Committee of the Laboratory Animal Center, Academy of Military Medical Sciences, China (IACUC-13-2016-001).

### Viruses, cells, and reagents

ZIKV strains GZ01 (GenBank: KU820898), FSS13025 (GenBank: JN860885), and DENV-2 (43 strain, GenBank: AF204178) were used in this study as mentioned previously^29^. BHK-21, HT-29, and SW620 cells were purchased from ATCC and cultured at 37 °C and 5% CO_2_ in DMEM, F12 or RPMI 1640 media, respectively. All media were supplemented with 10% FBS (ExCell Bio, Jiangsu), 100 units/ml penicillin, and 100 μg/ml streptomycin. 4G2 monoclonal antibody (mAb) targeting the ZIKV E protein was produced as previously described^29^. Mouse anti-Ifnar1 antibody (clone MAR1-5A3) was purchased from Leinco Technologies, Inc. (St. Louis, MO, USA).

### Sample collection and real-time quantitative PCR

Feces were collected immediately upon release and associated with individual mice. Fecal weight was recorded, and then the sample was suspended in 500 μl DMEM. 200 μl of the fecal suspension was used for RNA extraction. A volume of 150 μl PBS was used for anal lavage of each mouse. Total viral RNA isolated from mouse sera, cell supernatants, anal lavage or feces was extracted using the EasyPure Viral DNA/RNA Kit (TransGen Biotech, Beijing). Real-time quantitative PCR (qRT-PCR) was conducted to measure viral RNA copies. ZIKV primers were described previously^40^.

### ZIKV intra-anal infection in mice

Three to four-week-old male *Ifnar1*^−/−^ mice, C57 pregnant mice, or immunocompetent C57 mice were infected with ZIKV (strain GZ01/2016 or FSS13025) through the anal canal. Briefly, mice were inoculated with virus stock using a sterile animal feeding needle (20 G, 6 cm length, 1 mm diameter ball tip) carefully inserted into the anal canal to avoid trauma. The anal canal was tilted upwards for 5–10 min to help ensure infection. ZIKV RNA copies in sera were measured by qRT-PCR as described previously^29^. Four mice were euthanized for tissue microscopy or qRT-PCR analyses of ZIKV infection in the organs at the indicated time points.

### Plaque assay

BHK-21 cells were incubated with samples containing virus at 37 °C for 1 h. The plate was then washed once with PBS, and overlay media containing 2% FBS and 1% low melting temperature agar (Promega) was added for the plaque assay. Viral plaques were counted at 4 dpi after staining with crystal violet solution.

### In situ hybridization of ZIKV RNA

The assay was performed with an RNAscope 2.5 (Advanced Cell Diagnostics) according to the manufacturer’s instructions. Briefly, formalin-fixed paraffin-embedded (FFPE) tissue samples were deparaffinized for 60 min at 60 °C. Peroxidases in the samples were quenched with hydrogen peroxide at room temperature for 10 min. Slides were then heated in RNAscope Target Retrieval Reagents for 15 min and incubated in RNAscope Protease Plus before hybridization with probes for 30 min. The probe targeting ZIKV RNA was designed and synthesized by Advanced Cell Diagnostics (catalog no. 467871). Samples were counterstained with Gill’s hematoxylin and visualized with bright-field microscopy.

### ELISA assay

Briefly, 100 ng/well of ZIKV E protein was coated onto an ELISA plate. Mouse sera were diluted with PBS containing 1% BSA and added to the plate for 1 h at 37 °C. The plate was then washed three times using PBST and incubated with HRP-conjugated IgG or IgM for 1 h at 37 °C. After washing three times with PBST, the plate was incubated with TMB solution at 37 °C for 30 min. Stop solution was added to the wells for 3 min, and the OD450 was measured by a microplate reader.

### Luminex assay

The Cytokine 20-Plex Mouse Panel (LMC0006M, Thermo Fisher) and 25-Plex Human Panel (LHC0009, Thermo Fisher) was used to measure cytokines in mouse and human sera, respectively, which were diluted with assay dilution buffer. The assay was conducted using a Luminex200, and the results were analyzed by Luminex xPONENT according to the manufacturer’s protocol.

### RNA-seq analysis and GO analysis

Total RNA from HT-29 cells infected by ZIKV (GZ01 strain, MOI 0.5) for 24 h or mock-infected was isolated and used to analyze global mRNA transcription by RNA-seq. The Ribo-Zero™ Magnetic Gold Kit (Illumina, MRZG12324) was used to remove ribosomal RNA from 1 μg of the RNA sample, which was then cleaned by Agencourt RNAClean XP beads (Beckman Coulter, A63987). A volume of 5 μl of the eluate was used to create strand-specific RNA libraries using the NEBNext Ultra Directional RNA Library Prep Kit for Illumina (NEB, E7420L) according to the manufacturer’s instructions. The library quality was evaluated on the Agilent 2100 bioanalyzer and quantified by qPCR using the Universal KAPA Library Quantification Kit (KAPA Biosystems, KK4824). Libraries were sequenced on a HiSeq X10 instrument (paired ends 2*150 bp). Genes with a fold change > 1.2 and *p* < 0.05 between the ZIKV and mock groups were labeled as ZIKV-induced genes (ZIG). ZIG qRT-PCR primers were obtained from the Primer Bank (https://pga.mgh.harvard.edu/primerbank/). The mRNA level of each ZIG was analyzed using SYBR Green qPCR mix (TransGen Biotech, Beijing). Gene ontology analysis was performed using the GO enrichment analysis tool (http://geneontology.org/page/go-enrichment-analysis).

The raw sequence data reported in this paper have been deposited into the Genome Sequence Archive^41^ of the BIG Data Center, Beijing Institute of Genomics (BIG), Chinese Academy of Sciences, under accession number CRA000783, which is publicly accessible at http://bigd.big.ac.cn/gsa.

### Immunofluorescence assay (IFA)

HT-29 and SW620 cells were infected with ZIKV for 48 h and were fixed. Slices were blocked at room temperature for 1 h with 2% goat sera, 0.3% Triton X-100, and 5% BSA in PBS. The 4G2 mAb (5 μg/ml) was applied at room temperature for 1 h, and binding was visualized using FITC-conjugated secondary antibody. Cell nuclei were stained with DAPI (Sigma). Slices were imaged by fluorescence microscopy.

### ZIKV intra-anal infection of pregnant mice

Seven to eight-week-old male and female mice were mated, and the vaginal plugs of female mice were monitored. At E5.5 (embryonic day 5.5), 2 mg/mouse of anti-Ifnar1 antibody was administered to the pregnant mice to facilitate viral infection. These mice were then infected with 10^6^ PFU of the GZ01 strain through the anal canal at E6.5. The infected mice were euthanized to detect viral loads in the maternal sera and organs listed in Fig. 5b, as well as the fetal brain and placenta using qRT-PCR at E14.5, or to measure fetus sizes and viral loads in the placenta at E17.5.

### Statistical analysis

Data were analyzed by Prism software (GraphPad) or Microsoft Excel. All data are shown as the mean ± SEM if not otherwise indicated. For in vivo studies, the figures show data from a single representative experiment. *p < 0.05, **p < 0.01, ***p < 0.001, ****p < 0.0001, unpaired Student’s *t*-test.

## Electronic supplementary material

Supplemental Information
