# Supplementary material for: Zika virus shedding in the stool and infection through the anorectal mucosa in mice
Source: Emerg Microbes Infect. 2018 Oct 17;7:169. doi: 10.1038/s41426-018-0170-6 (PMC6193040; doi:10.1038/s41426-018-0170-6)
Supplement: Supplementary file 1 — Supplemental Information [file 41426_2018_170_MOESM1_ESM.docx]

**Supplemental Information:**


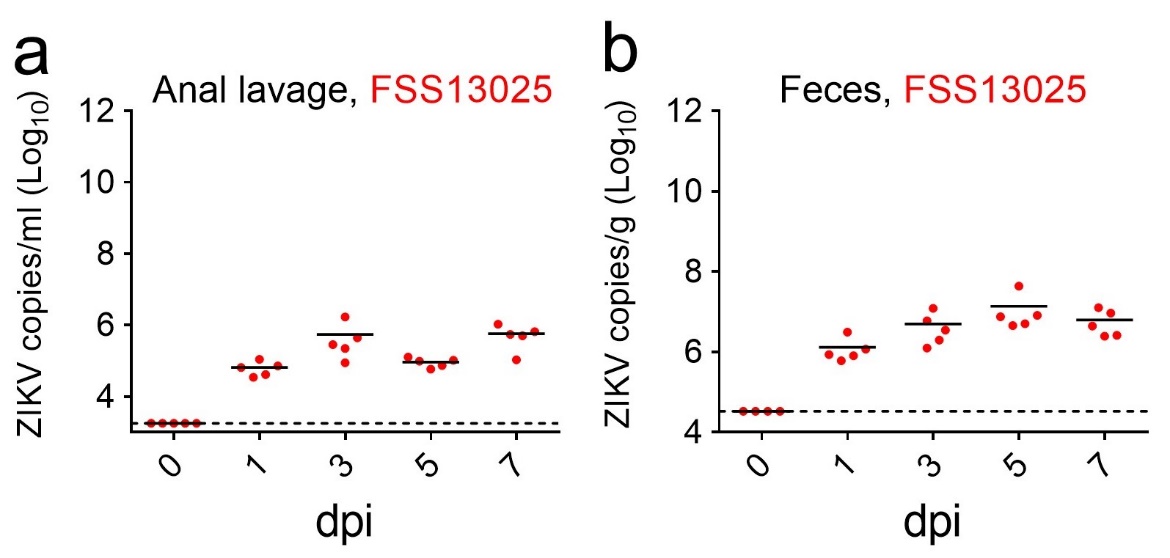


**Figure S1. Viral loads in anal lavage fluid and feces of *Ifnar1*^-/-^ mice**. 3-4 week-old male *Ifnar1^-/-^* mice were infected with 10^5^ PFU ZIKV (FSS13025 strain) by the *i.p.* route (n=5 for each group). Viral loads in anal lavage fluid (**a**) and feces (b) were detected by qRT-PCR.


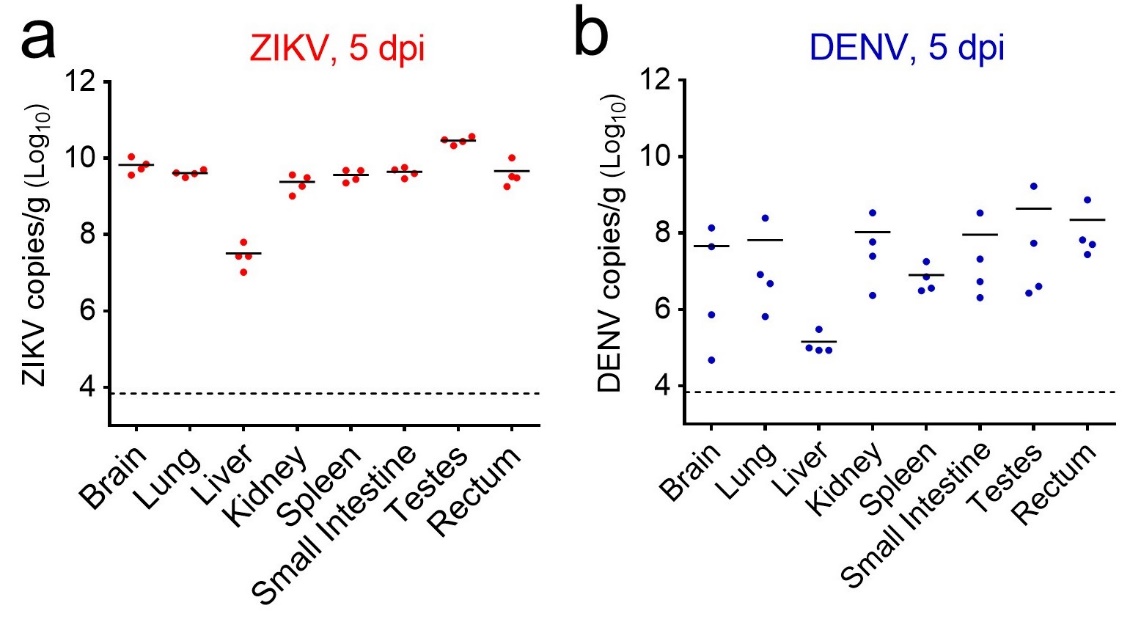


**Figure S2.** **Viral loads in selected organs of ZIKV- or DENV- infected *Ifnar1*^-/-^ mice**. *Ifnar1^-/-^* mice were infected with ZIKV **(a)** or DENV **(b)** by the *i.p.* route as shown in **Fig. 1** (n=4 per group). Viral loads in organs were detected by qRT-PCR.


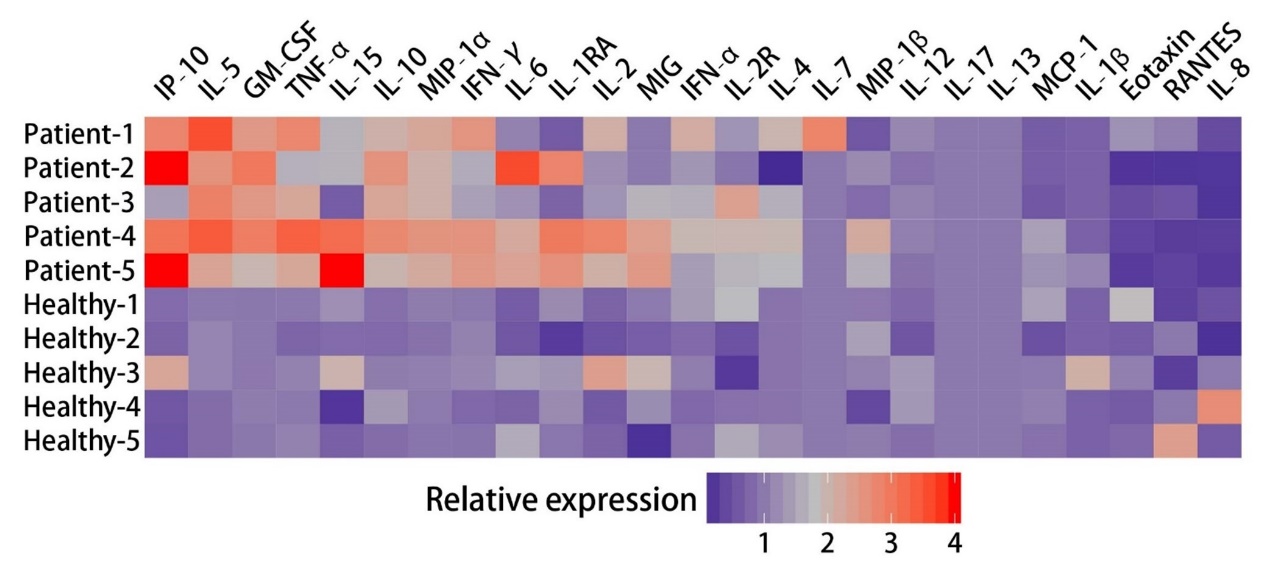


**Figure S3.** Cytokine levels in the serum of five healthy and five ZIKV-infected patients. Luminex assay was used to determine the cytokine level (n=5 per group). The average expression levels of different cytokines from the 5 healthy individuals were termed as “1”, respectively.


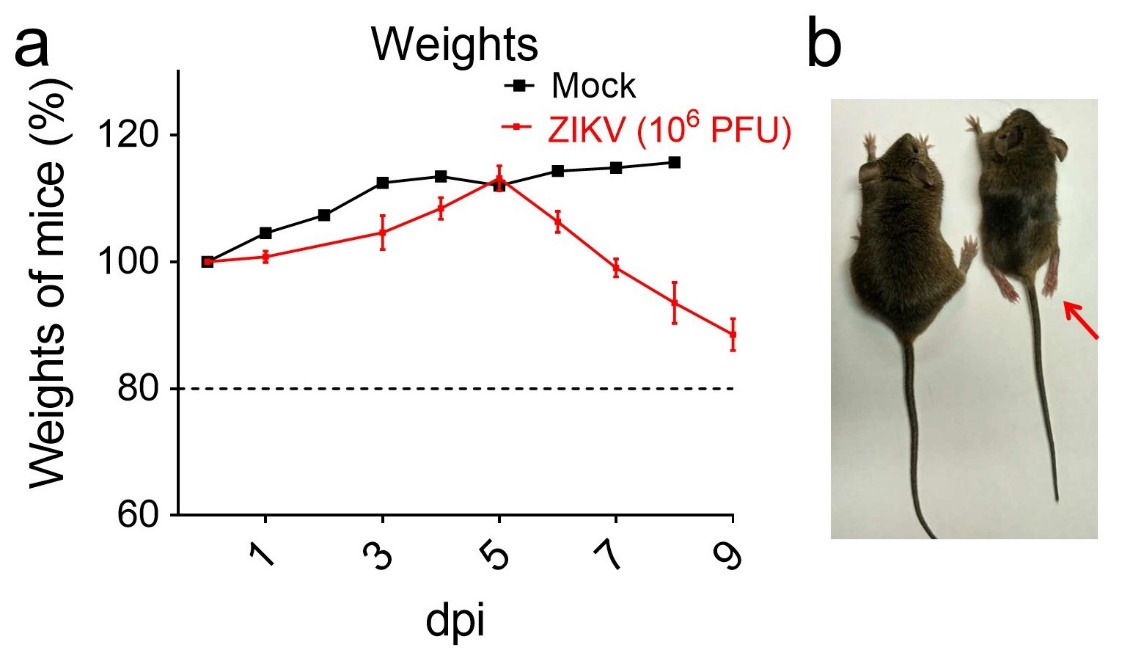


**Figure S4. ZIKV infection through *i.a.* route leads to body weight loss and neurological symptoms in *Ifnar1^-/-^* mice.** (**a**) Weights over time of *Ifnar1^-/-^*mice that were mock-infected (n=3) or infected *i.a*. with ZIKV (10^6^ PFU GZ01/mouse) (n=6), whose survival is graphed in **Fig. 3b**. (**b**) Comparison of uninfected (left) and ZIKV-infected (right) mice, with the latter displaying hindlimb paralysis (as indicated) from one representative experiment.


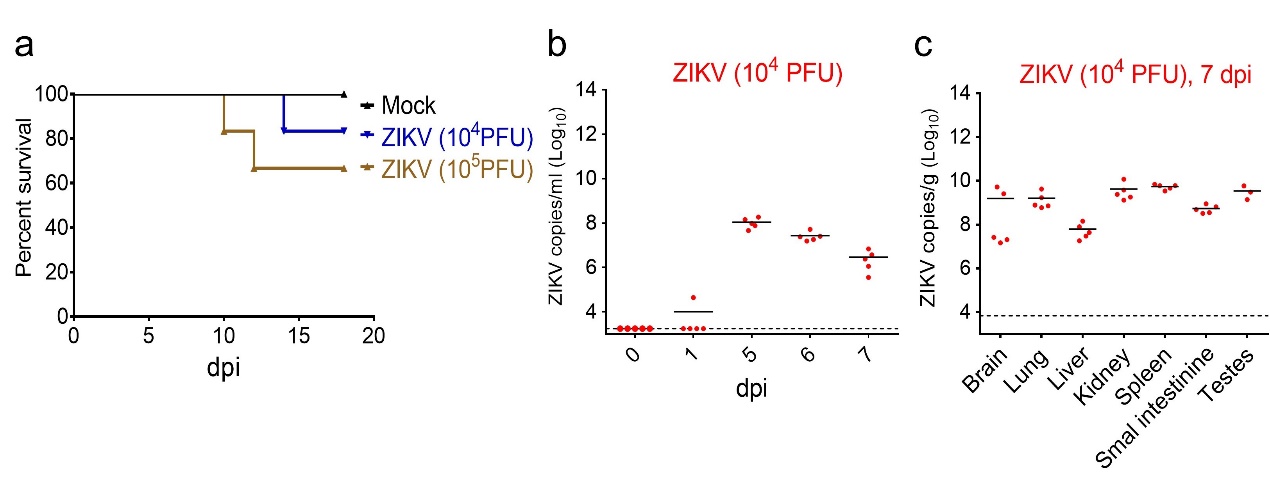


**Figure S5. Survival and viral loads in mice infected with ZIKV by the *i.a.* infection.** (**a**) Survival of *Ifnar1*^-/-^ mice infected with 10^4^ or 10^5^ PFU of GZ01 by the *i.a.* route (n=6 per group). ZIKV copies in sera (**b**) and organs (**c**) from *Ifnar1*^-/-^ mice infected with 10^4^ PFU of GZ01 by the *i.a.* route at the indicated time points (n=5 per time point, n=3 for testes).


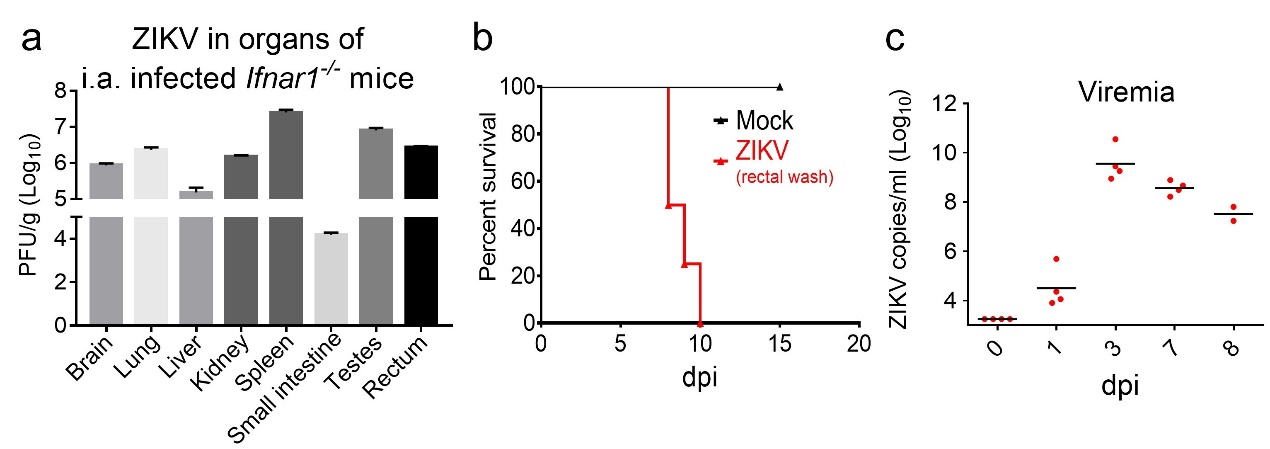


**Figure S6. Viral loads in organs and infectivity of rectal wash samples from ZIKV *i.a.-*infected *Ifnar1^-/-^* mice**. (**a**) The viral burden in organs from *Ifnar1^-/-^* mice that was measured by qRT-PCR in **Fig. 3d** was also quantified by plaque assay at 5 dpi, showing persistence of infectious virus. Data are shown as mean ± SD from two mice for each organ. (**b-c**) Survival (**b**) and viremia (**c**) of *Ifnar1^-/-^* mice infected with rectal wash samples by the *i.p.* route. To prepare wash samples, 2 cm of rectal tissue was isolated from *Ifnar1^-/-^* mice at 5 dpi, infected as shown in **Fig. 3b**, then washed with 500 μl of PBS. 200 μl of sample was used to infect naive *Ifnar1^-/-^* mice by the *i.p.* route. n=4 per group.


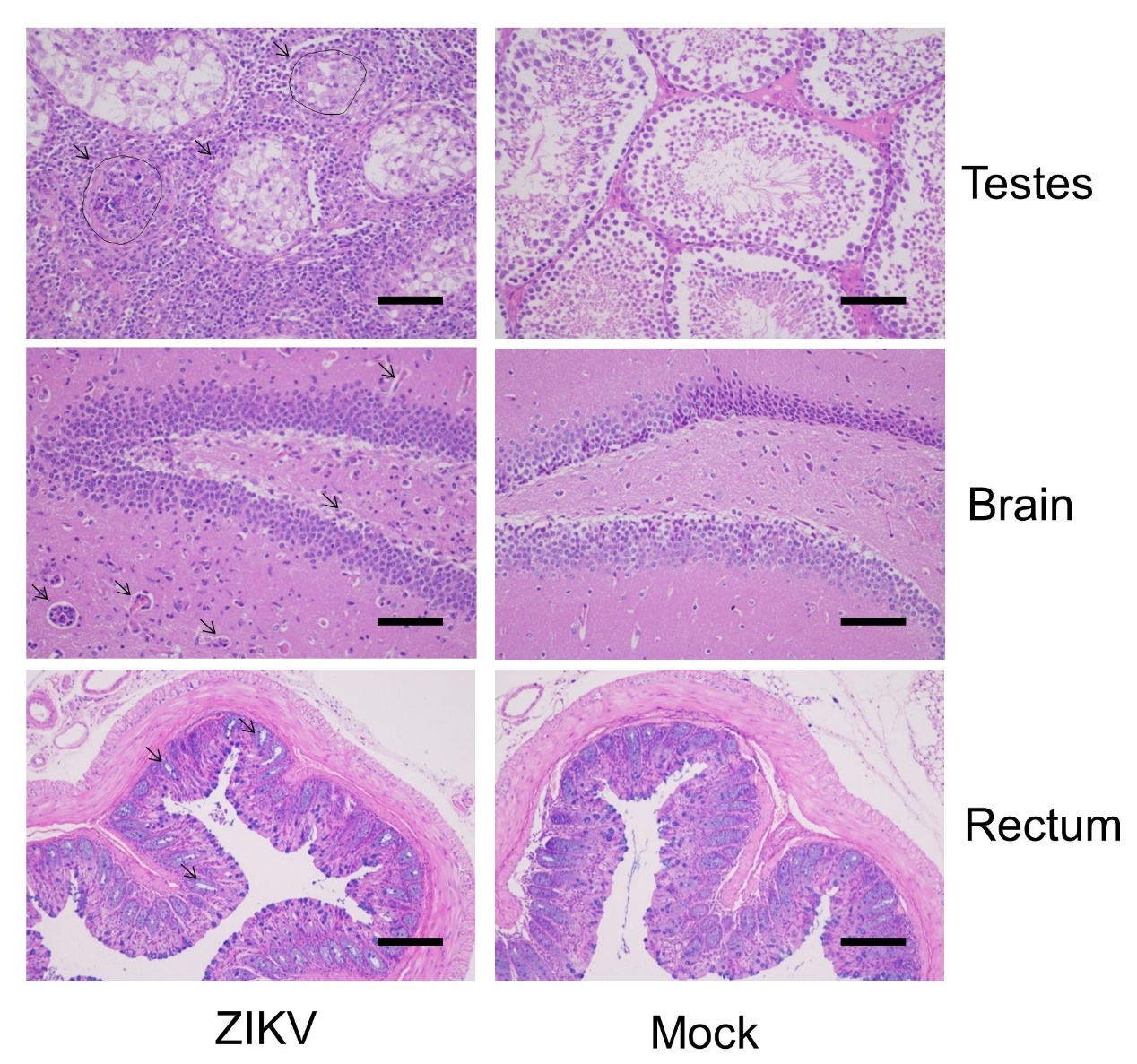


**Figure S7. Histopathological analysis of testes, brain and rectal tissue from the ZIKV *i.a-*infected *Ifnar1*^-/-^ mice.** Testicular damage and vascular cuffing and inflammatory cell infiltration in the brain and rectum of mice whose organ burden is graphed in **Fig. 3d**, magnification at 200X. The data are representative of two independent experiments with at least two animals per group at 15 dpi.


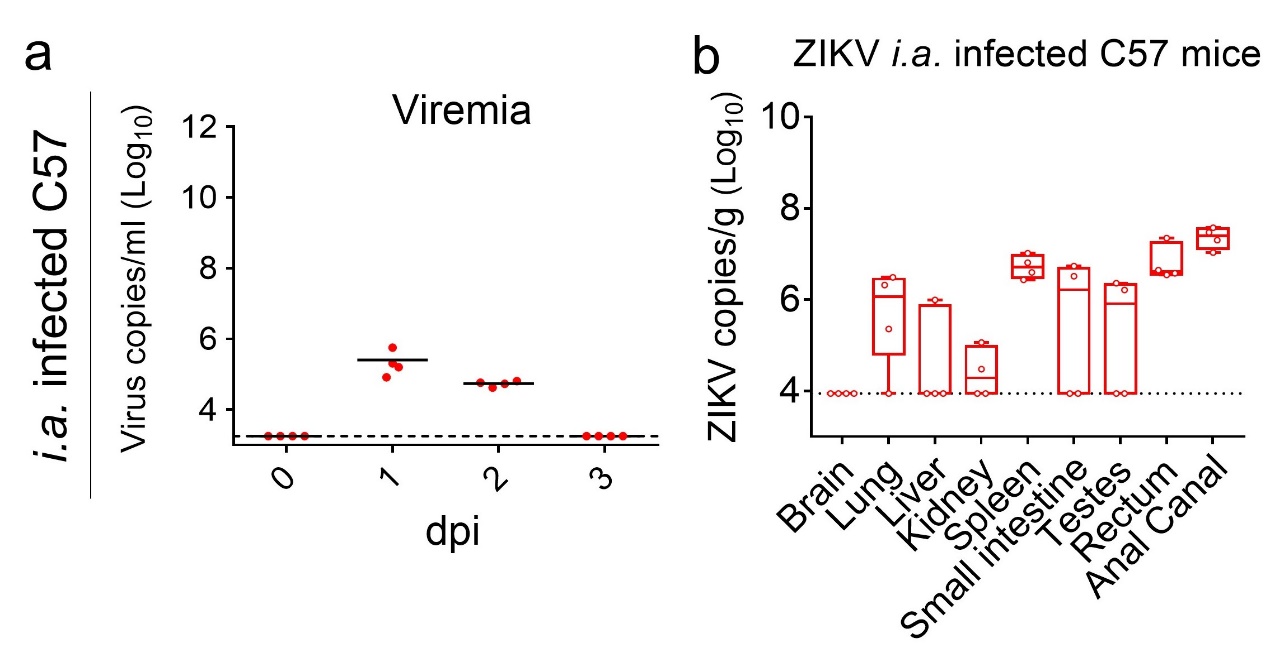


**Figure S8. The infectivity of ZIKV to immunocompetent mice through *i.a.* route.** (**a-b**) 3-4 week-old male C57 mice were infected i.a. with ZIKV (10^5^ PFU/mouse). ZIKV RNA in blood at 1-3 dpi (**a**) and organs (**b**) at 1 dpi were detected using qRT-PCR (n=4 per group).


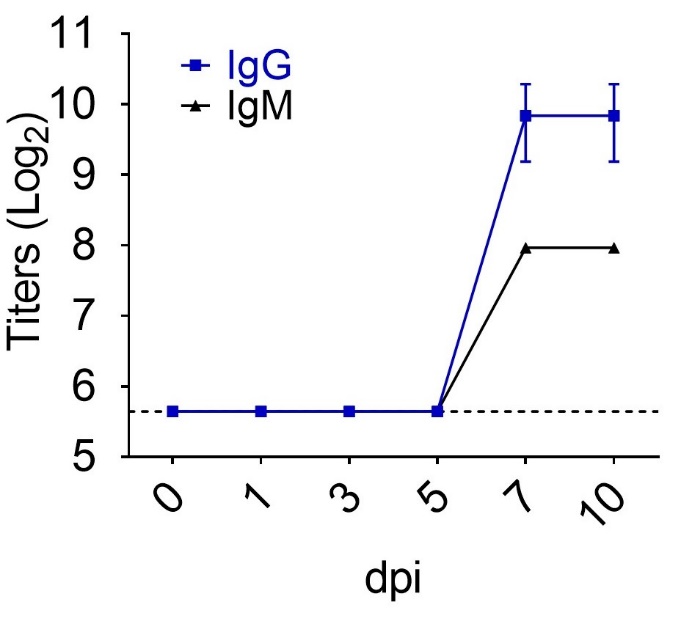


**Figure S9. Antibody response of ZIKV *i.a.* infection in *Ifnar1*^-/-^ mice.** Mice were infected with the GZ01 strain as shown in **Fig. 3b**. ZIKV-specific IgG and IgM in sera from ZIKV-infected mice at the indicated time points were measured by ELISA. Data was shown as mean ± SD (n=3 per time point).


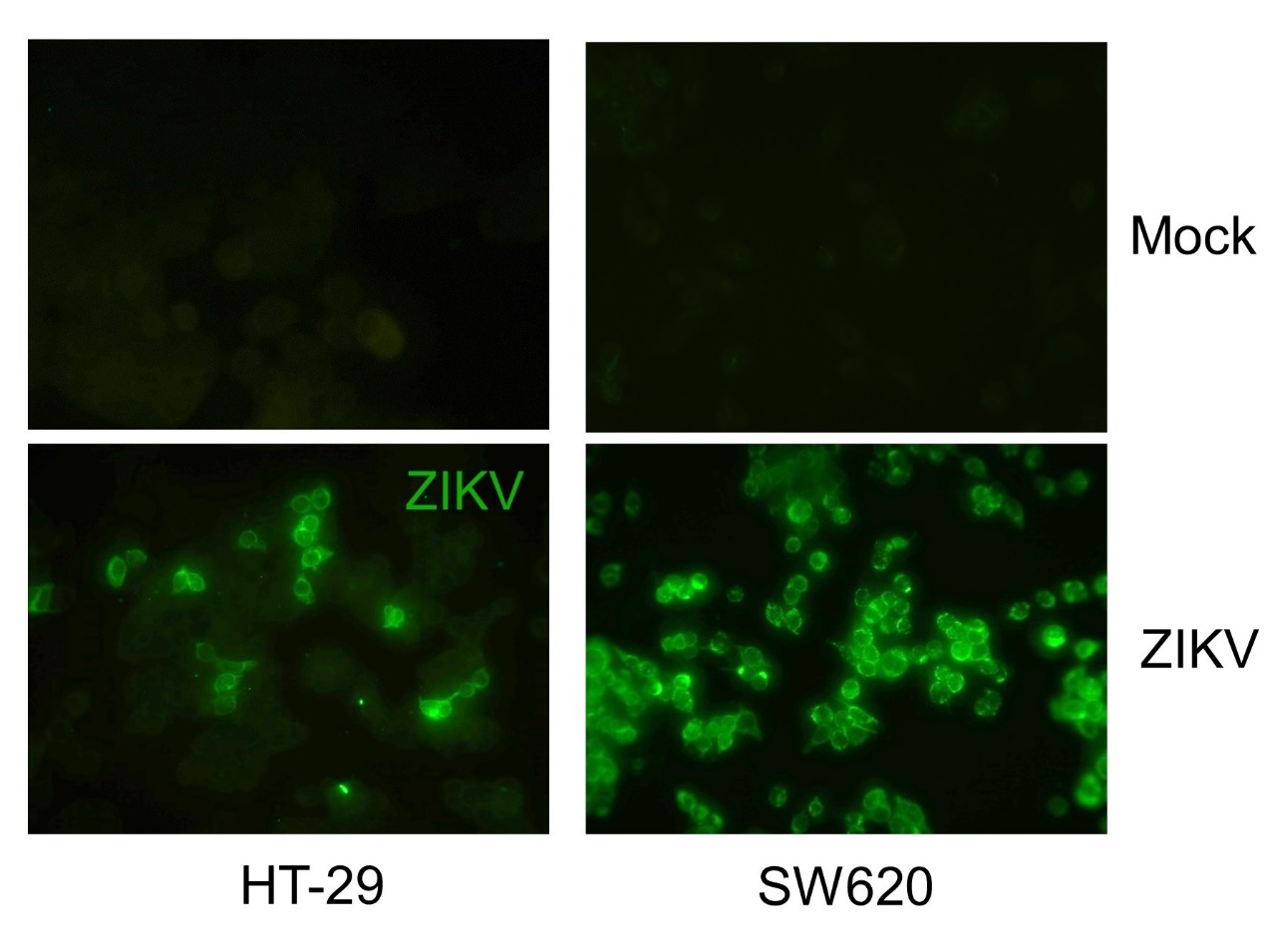


**Figure S10. ZIKV infection in human colon epithelial cells**. HT-29 and SW620 cells were infected with ZIKV (GZ01 strain, MOI 0.5) for 48 hrs. Infection efficacy of ZIKV was detected by IFA assay with 4G2 antibody targeting the ZIKV E protein. The data are representative of two independent experiments with at least two repeats per group.


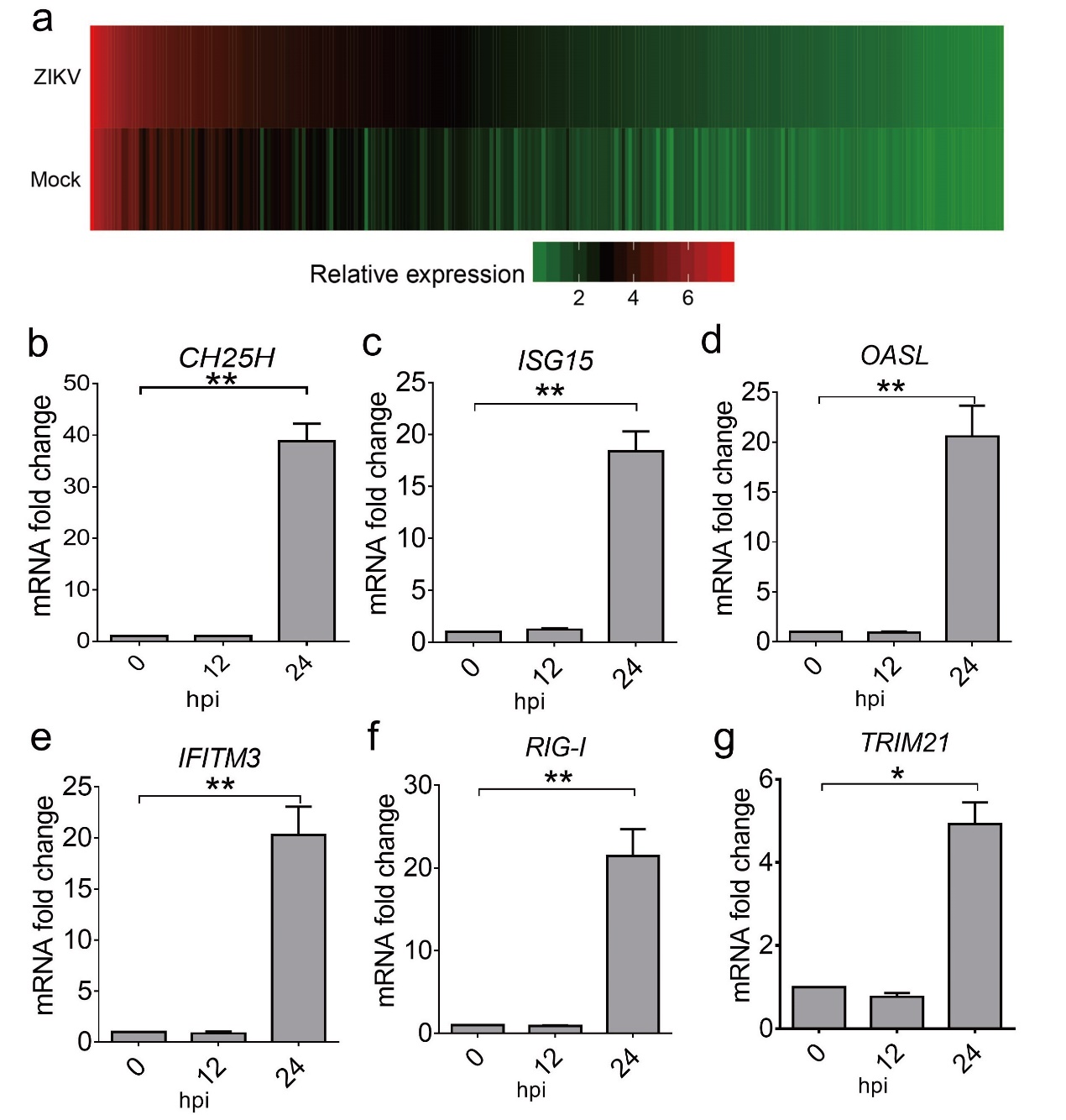


**Figure S11. Innate immune responses in human colon cells induced by ZIKV infection.** (**a-g**) HT-29 cells were infected with ZIKV at MOI=0.5 (**a**) or MOI=1 (**b-g**). (**a**) Relative expression of innate immune response genes in ZIKV-infected versus uninfected HT-29 cells was measured by RNA-seq. (**b-g**) Expression levels of *CH25H, ISG15, OASL, IFITM3, RIG-I* and *TRIM21* were quantified using qRT-PCR at 12 and 24 hpi. All data are shown as mean ± SEM. **p*<0.05, ***p*<0.01, unpaired student *t* test.
